# Stimulus conditions eliciting sneezing in response to bright light

**DOI:** 10.1007/s00221-024-06988-4

**Published:** 2025-02-01

**Authors:** Josef Trinkl, Lucien Bickerstaff, Stephan Munkwitz, Manuel Spitschan

**Affiliations:** 1 Max Planck Research Group Translational Sensory & Circadian Neuroscience, Max Planck Institute for Biological Cybernetics, Tübingen, Germany; 2 TUM School of Medicine and Health, Department Health and Sport Sciences, Chronobiology & Health, Technical University of Munich, Munich, Germany; 3 TUM Institute for Advanced Study (TUM-IAS), Technical University of Munich, Garching, Germany; 4 University Hospital and Faculty of Medicine, University of Tübingen, Tübingen, Germany

**Keywords:** Photic sneeze reflex, Sneezing, Light sensitivity, Stimulus-response relationship, Visual stimuli, Sensory stimulation

## Abstract

The photic sneeze reflex (PSR) is an involuntary sneezing response to bright light exposure, affecting approximately 25% of the population. Despite its long history in scientific literature, the underlying mechanisms remain unclear. Several theories, including optic-trigeminal summation, parasympathetic hypersensitivity, and parasympathetic generalization, have been proposed, but none have been conclusively validated. Reproducing the PSR reliably in a laboratory setting is crucial for understanding its neural underpinnings, yet the specific light parameters that trigger PSR are not well-defined. This mini-review aims to consolidate current knowledge on the light stimulus parameters (intensity, spectral composition, wavelength, duration, timing, spatial configuration) that elicit the PSR. A comprehensive literature search was conducted using MEDLINE (PubMed), Google Scholar, Google Books, and Google, employing terms related to photic sneezing in multiple languages. Articles from 2020 to 2024 were screened, resulting in 167 records, with seven studies focusing on stimulus parameters included in this review. The reviewed studies, including four case reports and three laboratory experiments, consistently support that bright light can induce sneezing in susceptible individuals. However, there is significant variability in the methodologies and outcomes, limiting comparability and indicating a need for systematic investigation. No study has yet examined the parametric relationship between light parameters and the PSR. The heterogeneity of methods and findings in the existing literature highlights the lack of standardized research on the specific light parameters that trigger the PSR. This review underscores the need for controlled experiments to clarify these relationships and improve our understanding of the underlying neural mechanisms. Existing research on photic sneezing stimulus parameters is fragmented and lacks systematic approaches. Future studies should focus on standardized, parametric investigations to elucidate the light-sensitive mechanisms of the PSR.

## Background

Sneezing in response to bright light is a common but poorly understood phenomenon, in which an estimated 25% of people sneeze when exposed to a very bright light stimulus, such as stepping outside of a building into bright daylight. In the scientific literature and common usage, this phenomenon has been termed the photic sneeze reflex (PSR) (Anderson and Rosenblith 1968; Leavitt 1984; Peroutka and Peroutka 1984; Whitman and Packer 1993; Keeton 1995), the solar sneeze reflex (Lang and Howland 1987), the photic sneeze syndrome (PSS) (Sasayama et al. 2019; 2018), autosomal dominant compulsive helio-ophthalmic outbursts of sneezing (ACHOO) (Subramanian and Shetty 2023; Collie et al. 1978; Morris 1987; 1989; Elwood et al. 1996; McKusick 2003; Dean 2012), photic sneezing or simply sun sneezing (this paper uses the term photic sneeze reflex, abbreviated as PSR). There is an extensive literature on the PSR dating back centuries, but a conclusive mechanistic explanation is thus far outstanding.

In the literature, different theories have been developed concerning the underpinnings of the PSR (Everett 1964; Askenasy 1990). One theory – the optic-trigeminal summation theory – posits a crosstalk between the second (CN II) and the fifth (CN V) cranial nerve at the level of the midbrain, resulting in referred sensations such as nose-prickling commonly experienced by photic sneezers. Various pathways are interconnected in the brainstem, representing a plausible site enabling integration of optic input and CN V (Schaller 2007; Semes et al. 1995; Kulas et al. 2017). A second theory – the parasympathetic hypersensitivity theory – posits that photic sneezers have a general hypersensitivity of their parasympathetic system and, therefore, enhanced excitability regarding stimuli. A third theory – the parasympathetic generalization theory – explains photic sneezing through a joint excitation of one branch of the parasympathetic nervous system, resulting in the co-activation of closely situated branches. For instance, a strong photic stimulus leads to pupillary constriction (CN III) which may be accompanied by lacrimation (CN VII) (Everett 1964). While these theories may, at face value, all be plausible, there is no solid anatomical or functional evidence base that would arbitrate between these distinct neuroanatomical theories.

Central to understanding a real-world phenomenon is the ability to reliably reproduce it in the laboratory. There have been very few attempts to induce photic sneezing through well-understood light stimuli. Light can be parametrized through various parameters, including intensity, spectral composition, wavelength, duration, timing, and spatial configuration. The parameters driving the PSR remain unclear. Under real-world conditions, the PSR is usually triggered by exposure to sunlight or daylight upon exiting a building. Both sunlight and daylight have distinct spectral (broad spectrum) and spatial properties (distinct, very bright light source for sunlight against a sizeable wide field of view for daylight) compared to electric light.

Understanding which light parameters trigger photic sneezing can offer insights into the underlying light-sensitive mechanisms. More specifically, any wavelength differences can be related to the spectral sensitivities of the known photopigments in the human eye (L, M, S cones, rods, and the melanopsin-containing intrinsically photosensitive retinal ganglion cells) (Spitschan 2019), and help delineating which stimulus conditions should be minimized to avoid the PSR.

Changes and differences in intensity of light may be used to modulate excitability of the photoreceptors, trigeminal nerve endings and subsequent neural structures (Nishino 2000; Babes et al. 2016; Huang and Szallasi 2017; Saunders et al. 2013). Since photic sneezing shares considerable similarities with potentially more pathogenic reflexes such as the trigeminocardiac reflex (Chowdhury et al. 2019), the oculocardiac reflex or the diving reflex (Buchholz et al. 2017) elucidating the underlying pathway of photic sneezing might be of value in their understanding. Parasympathetic dysfunction plays a crucial role in all trigeminal reflexes, in which sensory nerve endings send signals via the trigeminal ganglion forwarded along the short internuncial nerve fibres in the reticular formation where motor branches of the CN V nucleus are excited. (Buchholz et al. 2017; Schaller 2004) This eventually leads to many autonomic changes in sympathetic and parasympathetic adaptations like dilation of blood vessels and bronchia, mucosal secretion and cardiac output (Buchholz et al. 2017; Schaller et al. 2009). Additionally, understanding the pathophysiology of PSR may contribute to the comprehension of further pathologic syndromes like photophobia, a condition in which normal levels of light cause discomfort or pain for instance in patients with corneal abrasion or infections of the eyes or migraine (Lebensohn and Bellows 1934; Stringham et al. 2003; Patterson Gentile and Aguirre 2020; Do et al. 2009).

## Objective

This article reviews the current knowledge of stimulus parameters (intensity, spectral composition, wavelength, duration, timing, spatial configuration) eliciting the PSR.

## Methods

### Literature search and identification

To identify relevant papers on stimulus parameters eliciting the PSR, a pragmatic search strategy was deployed using MEDLINE (PubMed), Google Books, and simple Google searches. The search terms used were “photic sneeze”, “sun sneeze*”, “’bright light’ + sneeze”, and variants of this in English, German and French. These articles were then supplemented by both forward and backward literature searches on Google Scholar. This led to compiling a comprehensive bibliography on the PSR between 2020 and 2024 (first search 9 November 2020, first update 23 May 2021, second update 31 January 2024) comprising 167 records (n = 167 articles). The articles were screened by title, abstract, and full-text, and articles identifying stimulus parameters either in ‘incidental’ case reports or targeted laboratory studies were selected for inclusion in this narrative mini-review (n = 7 articles). A spot check on Web of Science revealed no additional articles (10 December 2024). The complete bibliography is available as a supplement to this article and on GitHub (https://github.com/tscnlab/TrinklEtAl_ExpBrainRes_2025).

### Author roles in literature search and review

MS completed the searches, compiled and updated the bibliography, and screened the articles. JT reviewed the articles and extracted the findings from each study.

## Results

### Individual studies

#### Freund (1904)

In early 20th century, Freund (1904) published a German-language self-report with data collected on himself. In his article, he describes the tangible sensations leading to a sneeze when changing from a shadowy into a bright area and experiments exposing potential candidate areas causing the PSR (eyes, face, nose, nasal mucosa). He further investigated the PSR under daylight illumination filtered by colored filter glasses. He concluded that light received by the eyes is the primary stimulus eliciting a photic sneeze, which can be facilitated by chemical stimulation of nasal mucosa by sniffing agents such as tobacco or soap powder. Photic irritation of nasal mucosa, even when the eyes were covered, could still elicit the reflex on some occasions. Light filtered through red and green glasses had stopped or could not trigger sneezing, whereas light seen through violet and blue filters appeared to increase the probability of eliciting the PSR.

#### Anderson & Rosenblith (1968)

In a survey investigating visual responses in human newborns, Anderson and Rosenblith (1968) kept track of the prevalence of photic sneezing. Their study tested the ability to fixate and follow a silver bicycle bell passing across the newborn’s field of view for between one and three minutes out of up to 30 min of total examination time. In instances when the bell could not elicit a response from the newborn, a penlight was used instead. The study comprises 275 infants, of whom 76 sneezed during the assessment procedure. Fifty-four babies sneezed once, eight sneezed twice, and three sneezed three times. No data on sneeze frequency were recorded for eleven cases. 36 of these 76 babies sneezed upon exposure to the bell. Within their article, they reported on a newborn with trisomy 13, an abnormal condition in which an additional chromosome 13 leads to multiple and complex organ dysfunction and severe neurodevelopmental dysplasia including microencephaly, microphthalmia or anophthalmia which often are associated with further neural deformations such as holoprosencephaly and defects of internal organs. The vision status of this newborn was unclear, but upon presentation of a flashlight shining into its left eye, it sneezed.

#### Lewkonia (1969)

Lewkonia (1969) reported a case about a single 47-year-old male individual who had experienced an injury in which potassium hydroxide was splashed into both eyes. His tendency to sneeze in quick succession was noted during a hospital stay when exposed to bright lights, particularly during examinations with the ophthalmoscope and slit lamp. In follow-up visits, the sneeze frequency was recorded, showing a decline with recovery. Upon questioning his susceptibility to sneezing at light, he recalled that he had sneezed after exposure to various bright lights since childhood. The author also briefly describes another patient at the hospital who sneezed during a slit lamp examination.

#### Morris (1989)

Morris (1989) describes a case of a single 55-year-old female individual who had been referred for routine electroencephalogram (EEG) because of a history of absence seizures and generalized tonic–clonic seizures since childhood. The seizures were triggered spontaneously and occasionally by sunlight. In the anamnesis, she stated that she had always been an “easy sneezer” but did not notice if light triggered sneezes. EEGs were recorded while the subject was awake and asleep. After three minutes of hyperventilation, photic stimulation was delivered by light flashes at a reported illuminance of 5328 lx and different temporal frequencies of square-wave flicker (1, 4, 8, 12, 15, and 20 Hz) for a minimum of five to six seconds each. The patient only sneezed at a frequency of 15 Hz on three out of four occasions (75%, 2–4 sneezes, inter-sneeze interval 2–4 s, latency ~ 9.9 s).

#### Breitenbach (1993)

Breitenbach et al. (1993) examined photic sneezing as a possible risk to combat pilots, examining a single male individual who had reported a life-long history of sneezing twice at sudden changes in light intensity. It was suspected that a specific wavelength could be the trigger. Monochromatic lights generated with light from an incandescent lightbulb passing through interference filters (430 [or 450], 532, and 560 nm; wavelengths were specified ambiguously) were examined, along with military and civilian aviation goggles and sunglasses. The viewing distance to the light source was adjusted to yield a corneal photopic illuminance at 350 lx across stimulus conditions. The participant sneezed in response to all stimuli invariably, indicating no wavelength preference at this illuminance.

#### Hydén & Arlinger (2009)

Hydén & Arlinger (2009) probed whether local neural activity can be measured in the nasal cavity in response to photic stimulation. Seven healthy individuals, including three known photic sneezers, were tested with stroboscopic light at 5 to 10 Hz or a halogen lamp in front of the subjects’ eyes. Two electrodes were placed in the left nasal cavity, and one reference electrode was placed on each cheek. Electrical activity was recorded using the evoked potential averaging technique, and tickling and sneezing were elicited several times, however the article does not describe the stimuli and how often they caused a sneeze. The participants susceptible to the PSR reported that the tickling appeared instantly after the stimulus was applied, but no corresponding reproducible electrical activity in the nose could be recorded from any participant.

#### Langer et al. (2010)

Langer et al. (2010) examined scalp EEG measurements in response to light presented on a CRT monitor. The study compared ten photic sneezers with ten control subjects (each group comprises five women and five men). They were asked about different aspects of photic sneezing before the experiments and about accompanied sensations perceived due to light stimulation between the trials. The experiments consisted of two blocks. First, the participants were exposed to a flickering checkerboard (2.5 Hz) on a computer monitor. In the second block, which was performed only with the group of photic sneezers, 250 ms light flashes at three brightness levels (inter-stimulus intervals 1000–2000 ms) were presented to evoke the PSR. The participants were asked to rate the occurrence and frequency of a tickling sensation. The results showed that the brightest flash did not consistently produce the strongest tickling sensation. The EEG results showed an increased excitability of the visual cortex, mainly in the cuneus, in photic sneezers. Furthermore, a stronger subjective nose prickling sensation was found to be correlated with stronger activation in the insula and the secondary somatosensory cortex and not the stimulus luminance themselves. The researchers concluded that the PSR is not a classical reflex at a brainstem or spinal cord level, but it likely involves the visual cortex and co-activation of somatosensory areas.

### Synthesis of findings

The studies discussed in this review comprise four case reports and three laboratory studies. An overview of the articles can be found in Table 1. They all support the phenomenon that bright lights induce sneezing in a large part of the population. Freund (1904) observed this trait in himself and accordingly set up experiments where he exposed distinct parts of his face to natural day- and sunlight, partly filtered through colored glass. Anderson and Rosenblith (1968) noted the phenomenon during a study on visual responses of human newborns. A significant proportion of the 275 babies were sneezing after looking at a shiny bicycle bell or into a flashlight. In the following years, few case reports were published in which the PSR was induced by artificial light sources, such as slit-lamp examination, flickering lights, or filtered light. Although these reports made interesting observations, they did not deliver sufficient authoritative results regarding intensity, wavelength, or other preferences (Morris 1989; Lewkonia 1969; Breitenbach et al. 1993). The study by Hydén and Arlinger (2009) used either a stroboscopic light or a halogen lamp in front of the seven participants’ eyes and was not able to measure electrical responses in the nasal mucosa. Langer et al. (2010) compared a group of ten photic sneezers to another ten people as a control group and concurrently measured tickling sensations evoked by light stimuli with EEG. Thus far, no study has systematically investigated parametric relationships between light parameters, sneezing probability, and/or light-evoked tickling sensations.

**Table 1:** Overview of studies reporting on stimulus characteristics for eliciting the PSR. “m” indicates male participants, “f” indicates female participants

Reference	Year	Type	Sample size	Stimulus	Findings
Freund (1904)	1904	Case report	n = 1 m	Natural light seen through different optical filters, sneezing partially facilitated by supplementary chemical agents	Sneezing occurs when entering a well-illuminated spot out of a dim area. Irritation of the eyes through light is more potent than irritation of nasal mucosa by light. Red and green filters suppressed sneezing, whereas blue and violet filter-glasses did not
Anderson and Rosenblith (1968)	1968	Laboratory study	n = 275 (138 f)	Shiny bicycle bell or flashlight presented for 1 to 3 min during visual examination	27% of infants sneezed at least once when stimulus was presented; distribution of photic sneezing in newborns doesn’t deviate markedly compared to general population according to sex and race
Lewkonia (1969)	1969	Case report	n = 1 m	Slit-lamp illumination during daily examination of the eyes	As the eyes became less irritable during convalescence, the sneezing became less numerous
Morris (1989)	1989	Case report	n = 1 f	Flicker (30 cm in front of patient’s eyes; 5328 lx at 15 Hz)	Photic sneezing was reproducible in 3 out of 4 occasions, indicating that the stimulus was adequate
Breitenbach, Swisher (1993)	1993	Case report	n = 1 m	Photoflood source; monochromator (430/450, 532, 560 nm); spectral filters/sunglasses; intensity was predetermined at 350 lx	No indication that any wavelength can elicit a sneeze better than the other
Hydén and Arlinger (2009)	2009	Laboratory study	n = 7	Stroboscopic light (5—10 Hz; 30 cm in front of the eyes); halogen lamp (12 V; 20 W; 2300 cd; 10 cm in front of the eyes)	Nose tickling appeared immediately after stimulus onset. No reproducible electrical activity in the nose could be recorded in any experiment
Langer, Beeli (2010)	2010	Laboratory study	n = 20 (10 f)	CRT screen (70 cm in front of subject’s eyes) displaying contrast-reversing checkerboards (every 400 ms); video projected light flashes onto a reflective aluminum board (230 cm in front of the eyes); constant brightness in 3 trials (200 repeats times for 250 ms)	Photic sneezers have a generally enhanced excitability of the visual cortex. Furthermore, it was demonstrated in this group that a stronger nose-prickling sensation was associated with activation in the insula and stronger activation of the secondary somatosensory cortex

## Discussion

### Current state of knowledge on the light parameters triggering the photic sneeze reflex

Light stimuli, both natural (from daylight) and artificial (from electric light sources), are potent triggers for sneezing in a significant portion of the population. Sneezing typically occurs a few seconds after light stimulation. Sensations commonly reported as tickling in the nose are not reflected in measurable electrical activity in the trigeminal-innervated nasal mucosa. These sensations appear to correlate with activity in associative cortical regions in individuals susceptible to the reflex. This finding suggests that the tickling sensation is a referred sensation, likely indicating the involvement of multiple neural structures in triggering the reflex. During light exposure, individuals exhibiting the PSR demonstrate heightened activity in the insula and secondary somatosensory cortex.

Unfortunately, no studies were able to generate conclusive mechanistic insights underlying photic sneezing. Developing a dose–response curve and characterizing of the spectral sensitivity for this phenomenon are areas of future research. Such approaches, utilizing wavelength differences, have been used to identify the photoreceptor mechanisms underlying, for example, melatonin suppression (Thapan et al. 2001; Brainard et al. 2001; 2008) or circadian phase shifting (St Hilaire et al. 2022).

### Heterogeneity of stimulus parameters and outcomes

The heterogeneity of the reviewed studies indicates that there have yet to be systematic investigations of the light parameters triggering the PSR. Each study employed different stimulus methodology or outcome measurements. This limits the comparability of the studies reviewed here and highlights the need for parametrized stimulus linking light exposure with PSR and related outcomes.

## Limitations of this review

Despite the comprehensive search strategy across multiple search platforms and languages, it is conceivable that relevant scientific literature was missed. There may be unpublished results or data in the grey literature investigating stimulus conditions eliciting the PSR. Due to the heterogeneity of the literature and associated search terms, it is unclear whether a different search strategy could have yielded a more extensive selection of articles.

## Conclusion

This article has reviewed the literature on stimulus parameters causing photic sneezing. For this purpose, we used a pragmatic search strategy using a variety of search engines. Articles were screened and selected for content on stimulus parameters, then reviewed and extracted for the findings. A total of seven articles were included. The literature on photic sneezing is very heterogeneous, prompting further systematic investigations on the parametric relationship between light exposure and photic sneezing to better understand the neural mechanisms. Therefore, stimuli should be systematically varied in wavelength, spectral composition, intensity, duration, in timing, frequency and spatial configuration. The physiological underpinnings of PSR may ultimately be illuminated and help comprehend pathogenic conditions like photophobia and/or similar trigeminal reflexes.

## Data Availability

All data and materials can be downloaded under a Creative Commons license (CC-BY) from our GitHub repository at https://github.com/tscnlab/TrinklEtAl_ExpBrainRes_2025.
